# Survival Analysis of Single Large (>5 cm) Hepatocellular Carcinoma Patients: BCLC A versus B

**DOI:** 10.1371/journal.pone.0165722

**Published:** 2016-11-15

**Authors:** Yuri Cho, Dong Hyun Sinn, Su Jong Yu, Geum Youn Gwak, Ji Hoon Kim, Yang Jae Yoo, Dae Won Jun, Tae Yeob Kim, Hyo Young Lee, Eun Ju Cho, Jeong-Hoon Lee, Yoon Jun Kim, Jung-Hwan Yoon

**Affiliations:** 1 Department of Internal Medicine and Liver Research Institute, Seoul National University College of Medicine, Seoul, Republic of Korea; 2 Department of Internal Medicine, CHA Gangnam Medical Center, CHA University, Seoul, Republic of Korea; 3 Department of Medicine, Samsung Medical Center, Sungkyunkwan University School of Medicine, Seoul, Republic of Korea; 4 Department of Internal Medicine, Korea University college of Medicine, Guro Hospital, Seoul, Republic of Korea; 5 Department of Internal Medicine, Hanyang University College of Medicine, Seoul, Republic of Korea; 6 Institute of Medical Science, Hanyang University, Seoul, Republic of Korea; Chang Gung Memorial Hospital Kaohsiung Branch, TAIWAN

## Abstract

**Background & Aims:**

Single large (>5 cm) hepatocellular carcinoma (HCC) is classified as Barcelona Liver Clinic (BCLC) stage early stage (A). Yet, controversies exist whether single large HCC can be considered as early stage. We have analyzed long-term outcome to see which stage is appropriate for these patients.

**Methods:**

From 2005 to 2006, 1,546 consecutive patients who were newly diagnosed as HCC (BCLC A or B) at four tertiary hospitals in Korea were analyzed. BCLC A was sub-classified into A1 (single 2–5 cm), A2 (2–3 nodules ≤3 cm), and A3 (single >5 cm). BCLC B1 included patients beyond-Milan criteria, and within up-to-7 criterion. Survival prediction between subgroupings (1: A1 + A2 + A3 *vs*. B1 and 2: A1 + A2 *vs*. A3 + B1) was compared based on c-index and Akaike information criterion (AIC).

**Results:**

The 5-year overall survival (OS) rate was 62.3, 58.6, 36.8, and 42.0% for A1, A2, A3 and B1, respectively. In multivariate Cox-regression analysis, OS was significantly different between A3 + B1 *vs*. A1 + A2 (hazard ratio [HR] 1.85; *P*<0.001), but not between A1 + A2 + A3 *vs*. B1 (HR 1.19; *P* = 0.258). For A3, surgical resection showed superior OS over transarterial chemoembolization. Survival prediction was superior in subgrouping 2 (AIC 5727.2; c-index 0.652) than subgrouping 1 (AIC 5766.3; c-index 0.619) even after inverse probability weighting.

**Conclusions:**

This large scale long-term follow-up data shows that single large tumor should be considered as intermediate stage in terms of prognosis. However, in terms of treatment, resection might be the first line treatment option.

## Introduction

Hepatocellular carcinoma (HCC) is ranked as the fifth most common cancer and the second leading cause of cancer-related death worldwide [1, 2]. The incidence of HCC continues to be high in endemic areas, including Korea [3]. Clinical staging for cancer provides a guidance to predict survival outcome and to decide optimal treatment strategies [4]. The prognosis and treatment for patients with HCC depend not only on HCC stage but also on the state of liver function.

Barcelona Clinic Liver Cancer (BCLC) staging had been developed in 1999 on the basis of identification of prognostic factors for both liver cancer and hepatic function [5]. Single HCC <5 cm or 3 tumors <3 cm were classified as BCLC stage A at this time. However, single large (>5 cm) HCC was ambiguous to be classified. The original BCLC staging system has been externally validated in different clinical settings [6–8] and is endorsed by the American Association for the Study of Liver Diseases (AASLD), and the European Association for the Study of Liver (EASL) [9, 10].

The original BCLC staging system has been updated in 2011 [10]. It was regarded that single tumor reflects a more benign biological behavior. For this reason, single tumors beyond 5 cm are classified as BCLC stage A in the updated BCLC staging system. However, other studies have classified single large HCC as BCLC stage B, because large tumor size is regarded as an independent risk factor for recurrence and mortality [11, 12]. BCLC classification of single large HCC still remains controversial.

Patient with intermediate-stage disease (BCLC stage B) lies between the definitions of early and advanced HCC consisting of heterogenous patients with Child–Pugh (CP) class A and B liver function with large/multifocal HCC. There was a report which proposed four substages of intermediate HCC patients, B1 to B4 [13]. HCC patients with BCLC stage B1 showed favorable prognosis as comparable with that of patients with BCLC stage A. However, the current BCLC staging system does not provide any subgroup stratification for BCLC stage B patient population.

Actually, single large (>5 cm) HCC is beyond the indication of radiofrequency ablation (RFA) or liver transplantation (LT) according to the BCLC treatment guideline. Moreover, a patient with single large HCC and also portal hypertension, who is not indicated for surgical resection, is classified as BCLC stage A according to the current BCLC staging system. If a patient with single large HCC has different prognosis as compared to early stage (BCLC A) HCC patient, it might be beneficial to classify those patients into BCLC stage B1 to choose proper treatment option in terms of overall survival (OS) benefit [14]. Therefore, we have performed survival analysis of patients with single large (>5 cm) HCC to confirm the identity of single large (>5 cm) HCC by comparing survival prediction when it is classified as BCLC A *vs*. B.

## Patients and Methods

### Study Population

From January 2005 to December 2006, all the consecutive patients who had been newly diagnosed as HCC with BCLC A or B at four tertiary hospitals in Korea (Seoul National University Hospital, Seoul, Korea; Samsung Medical Center, Seoul, Korea; Korea University Guro Hospital, Seoul, Korea; Hanyang University Medical Center, Seoul, Korea) was retrospectively evaluated in this study. HCC was diagnosed according to the non-invasive criteria of the AASLD [9]. The investigations will include taking of patient history of the present illness, laboratory data, serum α-fetoprotein (AFP) levels, serum viral hepatitis markers, and radiological evaluations. BCLC stage A is sub-classified into A1 (single 2–5 cm), A2 (2–3 nodules ≤3 cm), and A3 (single >5 cm), which is different from that of Llovet’s in 1999 [15]. BCLC stage B was sub-classified according to the proposed BCLC B subclassification (B1–B4) [13]. BCLC B1 included patients with compensated cirrhosis, Eastern Cooperative Oncology Group (ECOG) performance status 0 (completely preserved), and large multinodular, but still not bulky (beyond Milan criteria [16], within up-to-7 criterion [17]). Survival prediction between subgroupings was compared between subgrouping 1 (A1 + A2 + A3 *vs*. B1) and subgrouping 2 (A1 + A2 *vs*. A3 + B1). For additional survival analysis, we took patients with BCLC B2 (CP class A, beyond up-to-7 criterion), B3 (CP score of 7, beyond up-to-7 criterion), and B4 (CP score 8–9, beyond up-to-7 criterion) into consideration.

In addition, to evaluate the effect of BCLC staging system on survival gain from treatment according to the BCLC guideline, subgroup analysis was performed according to the initial treatment modality (surgical resection *vs*. transarterial chemoembolization). Moreover, to evaluate the effect of the presence of liver cirrhosis (LC) on the discrimination function of each staging systems for OS, subgroup analysis was performed according to the presence of LC. The presence of LC was evaluated by the presence of any of the following clinical indicators of cirrhosis: thrombocytopenia (<150,000 platelets per μL), cirrhotic configuration of the liver (nodular liver surface or caudate lobe hypertrophy) and/or splenomegaly confirmed in imaging studies, or the presence of varices (abnormally enlarged veins, detected by upper endoscopy or cross-sectional images) [18]. Patients who underwent liver transplantation for HCC treatment were excluded.

### Statistical Analysis

OS was measured from date of enrollment until death from any cause. Survival prediction between subgroupings was compared based on the followings: Kaplan-Meier analysis as well as log-rank test; c-statistic; Akaike information criterion (AIC). Cox proportional hazard regression analysis was performed to find significant predictive factor for OS. Statistical analysis was performed with SPSS version 18.0 (SPSS Institute, Inc., Chicago, IL, USA) and R language version 3.1.1 (R Foundation for Statistical Computing, Vienna, Austria). To minimize selection bias in our observational study, inverse probability weighting (IPW) was used. IPW is a statistical technique for calculating statistics standardized to a population different from that in which the data was collected [19]. Propensity scores (PS) were calculated by generating a logistic regression model to estimate the average causal effect. We predicted the probability of each patient on the basis of the variables. PS was used to balance covariates across each group. After IPW (propensity score weight) were created, the groups were then balanced by means of IPW. A *P*-value of < 0.05 was considered statistically significant.

The study protocol conformed to the ethical guidelines of the World Medical Association Declaration of Helsinki and was approved by the Institutional Review Board of Seoul National University Hospital (IRB No. 1506-007-676). This study is a retrospective analysis. Therefore, we could not obtain the written informed consents. The data were analyzed anonymously.

## Results

### Baseline characteristics

During the study period, 1,546 consecutive HCC patients with BCLC A or B were newly diagnosed and staged at four tertiary hospitals (619 from Seoul National University Hospital; 693 from Samsung Medical Center; 155 from Korea University Guro Hospital; 79 from Hanyang University Medical Center). Patients with BCLC A1 (n = 772), A2 (n = 222), A3 (n = 251), B1 (n = 96), B2 (n = 89), B3 (n = 92) or B4 (n = 24) were analyzed.

The median age was 57.9 years (range, 28.8–96 years) and 1,145 patients (74.1%) were male (Table 1). Chronic hepatitis B was the predominant cause of HCC (1,117 of 1,546, 72.3%) and 222 patients (14.4%) had chronic hepatitis C, and 12 patients had both. LC was found in 72.1% (1,114 of 1,546) of patients. Most of them (905 of 1,114; 81.2%) were with CP class A. Median tumor size was 3.1 cm (range, 0.5–20.0), and median number of nodules was one (range, 1–5). As a first HCC treatment, transarterial chemoembolization (TACE) was performed for 785 patients (50.8%), surgical resection for 441 (28.5%), and RFA or percutaneous ethanol injection therapy (PEIT) for 320 (20.7%). Median follow-up period was 3.1 years (range, 0.1–10.5). During the follow-up period, 798 patients died.

**Table 1:** Baseline characteristics.

Clinical characteristics	n = 1,546
Age, years	57.9 (28.8–96)
Male	1,145 (74.1%)
Etiology of HCC	
HBV	1117 (72.3%)
HCV	222 (14.4%)
HBV + HCV	12 (0.8%)
Alcohol	61 (3.9%)
Others*	134 (8.7%)
Serum ALT, IU/L	42 (5–436)
Serum albumin, g/dL	3.7 (2.1–5.4)
Serum total bilirubin, mg/dL	0.9 (0.1–13.3)
Serum creatinine, mg/dL	1.0 (0.5–2.3)
Serum platelet count, ×10^3^/μL	132 (22–468)
PT-INR	1.1 (0.8–2.4)
Serum AFP, ng/mL	46.9 (0.8–570,000)
Serum PIVKA-II, mAU/mL	47 (8–22,356)
Maximal tumor size, cm	3.1 (0.5–20.0)
Number of nodules	1 (1–5)
BCLC subclassification	
A1 (single 2–5 cm)	772 (49.9%)
A2 (2–3 nodules ≤3 cm)	222 (14.4%)
A3 (single >5 cm)	251 (16.2%)
B1 (beyond MC, within up-to-7)	96 (6.2%)
B2 (beyond up-to-7, CP score 5–6)	89 (5.8%)
B3 (beyond up-to-7, CP score 7)	92 (6.0%)
B4 (beyond up-to-7, CP score 8–9)	24 (1.6%)
Presence of LC	1114 (72.1%)
CP class A	905 (58.5%)
CP class B	238 (15.4%)
Initial treatment modality	
TACE	785 (50.8%)
Surgical resection	441 (28.5%)
RFA or PEIT	320 (20.7%)

Abbreviation: HCC, hepatocellular carcinoma; HBV, hepatitis B virus; HCV, hepatitis C virus; NAFLD, non-alcoholic fatty liver disease; PBC, primary biliary cirrhosis; ECOG, Eastern Cooperative Oncology Group; ALT, alanine aminotransferase; PT-INR, prothrombin time-international normalized ratio; AFP, alpha-fetoprotein; PIVKA-II, prothrombin induced by vitamin K absence-II; BCLC, Barcelona clinic liver cancer; CP, Child-Pugh; LC, liver cirrhosis; TACE, transarterial chemoembolization; RFA, radiofrequency ablation; PEIT, percutaneous ethanol injection therapy

*NAFLD, PBC, Wilson’s disease, etc.

### Survival according to subgrouping 1 (A1 + A2 + A3 vs. B1) vs. 2 (A1 + A2 vs. A3 + B1)

**Fig 1:**
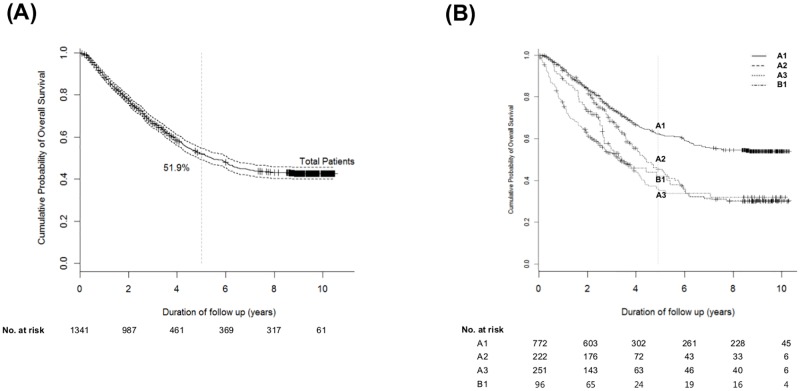
Cumulative overall survival rate of patients with BCLC A or B1. (A) Cumulative overall survival rate of patients with BCLC A or B1 (B) Respective cumulative overall survival rates of patients with BCLC A1, A2, A3 and B1.

**Fig 2:**
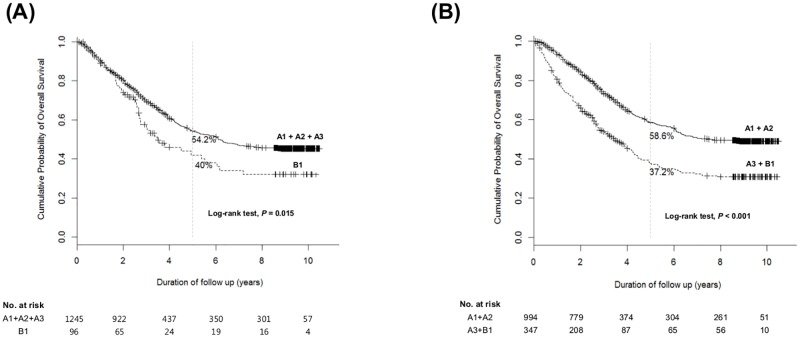
Cumulative overall survival rate of each subgrouping. (A) Overall survival rates according to subgrouping 1 (A1 + A2 + A3 *vs*. B1) (B) Overall survival rates according to subgrouping 2 (A1 + A2 *vs*. A3 + B1).

The 5-year overall survival (OS) rate of all the patients with A or B1 was 51.9% (Fig 1A). The 5-year OS was 62.3, 58.6, 36.8, and 40.0% for A1, A2, A3 and B1, respectively (Fig 1B). The 5-year OS rate of A3 was significantly lower than that of A1 and A2, respectively (both *P*<0.05). There was no significant difference in OS between A3 and B1 by log-rank test (HR 0.83; 95% CI 0.60–1.15; *P* = 0.264). Kaplan-Meier curves are presented in Fig 2A and 2B according to subgrouping 1 and 2, respectively. By log-rank test, subgrouping 2 (*P*<0.001) discriminated OS superiorly as compared to subgrouping 1 (*P* = 0.015). In subgrouping 1, 5-year OS of patients including A1 + A2 + A3 was 54.2%, while that of patients with B1 was 40% (Fig 2A). While, in subgrouping 2, 5-year OS of patients including A1 + A2 was 58.6%, while that of patients with A3 + B1 was 37.2% (Fig 2B).

**Fig 3:**
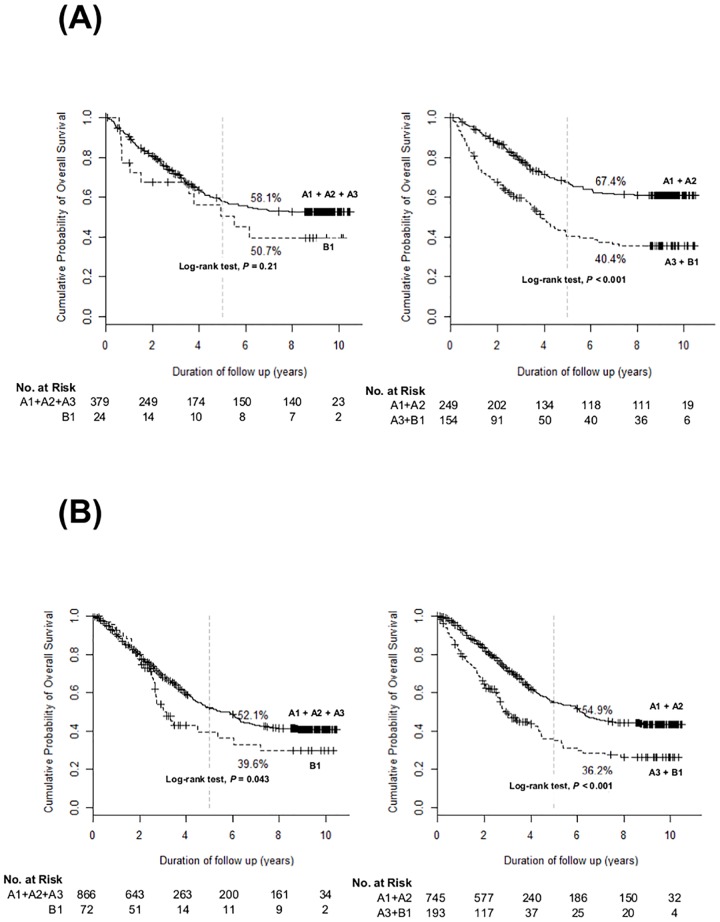
Overall survival of patients with liver cirrhosis (A) and those without liver cirrhosis (B) at baseline.

We performed subgroup analyses according to the presence of LC. As shown in Fig 3A for the patients without LC, only subgrouping 2 showed significantly different OS between A1 + A2 *vs*. A3 + B1 (*P*<0.001 by log-rank test), not subgrouping 1 (A1 + A2 + A3 *vs*. B1, *P* = 0.21). Among the patients with LC (Fig 3B), both subgrouping 1 (*P* = 0.043 by log-rank test) and subgrouping 2 (*P*<0.001 by log-rank test) significantly discriminated OS of each grouping.

**Fig 4:**
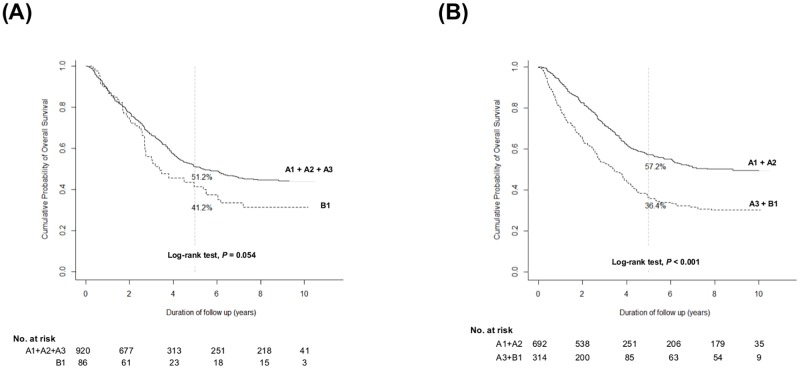
Cumulative overall survival rate of each subgrouping after IPW. (A) Overall survival rates according to subgrouping 1 (A1 + A2 + A3 *vs*. B1) after IPW (B) Overall survival rates according to subgrouping 2 (A1 + A2 *vs*. A3 + B1) after IPW.

**Fig 5:**
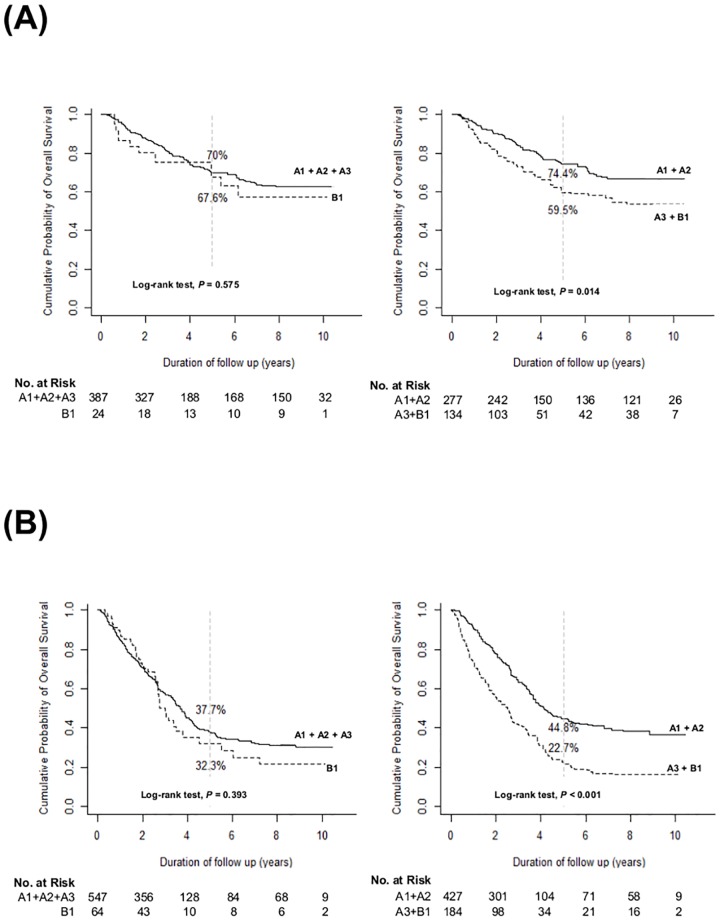
Overall survival of patients who underwent surgical resection (A) and those who underwent transarterial chemoembolization (B) as an initial HCC treatment.

We performed IPW with the covariates including age, sex, the etiology of HCC, ECOG, and the presence of LC to make more balanced subgroups (A1, A2, A3 and B1). Even after IPW, subgrouping 2 (*P*<0.001) showed superior discrimination function on OS by log-rank test (Fig 4) over subgrouping 1 (*P* = 0.054). In subgroup analyses, according to the initial treatment modality, subgrouping 2 also showed superior discrimination function by log-rank test over subgrouping 1. For the patients who underwent surgical resection (Fig 5A) as an initial HCC treatment, subgrouping 2 (*P* = 0.014 by log-rank test) was superior in discriminating OS over subgrouping 1 (*P* = 0.575 by log-rank test). For the patients who received TACE as an initial HCC treatment (Fig 5B), subgrouping 2 (*P*<0.001) was also superior in discriminating OS over subgrouping 1 (*P* = 0.393).

### Cox proportional hazard regression analysis for overall survival

To confirm that subgrouping 2 has significant predictive ability for OS, Cox proportional hazard regression analyses were performed. In univariate analysis, age (hazard ratio [HR] 1.01; 95% confidence interval [CI] 1.01–1.02; *P*<0.001), the presence of LC (HR 1.21; 95% CI 1.04–1.42; *P* = 0.016), initial treatment modality (TACE *vs*. surgical resection; HR 2.98; 95% CI 2.47–3.59; *P*<0.001), subgroup 1 (HR 1.44; 95% CI 1.07–1.92; *P* = 0.016) and subgroup 2 (HR 1.89; 95% CI 1.59–2.26; *P*<0.001) were the significant predictive factors for OS (Table 2). In multivariate Cox regression analysis, OS was significantly different between subgrouping 2 (HR 1.85; *P*<0.001), but not subgrouping 1 (HR 1.19; *P* = 0.258).

**Table 2:** Cox proportional hazard regression analysis for overall survival.

Variable	Univariate analysis		Multivariate analysis^1^		Multivariate analysis^2^	
	HR (95% CI) *P*		HR (95% CI) *P*		HR (95% CI) *P*	
Sex*	0.88 (0.74–1.04)	0.125				
Age	1.01 (1.01–1.02)	<0.001	1.01 (1.00–1.02)	0.178	1.01 (1.00–1.02)	0.183
Presence of LC	1.21 (1.04–1.42)	0.016	1.04 (0.84–1.29)	0.706	0.93 (0.75–1.15)	0.511
Initial treatment modality^†^	2.98 (2.47–3.59)	<0.001	2.71 (2.17–3.38)	<0.001	2.74 (2.19–3.42)	<0.001
Subgrouping 1^‡^	1.44 (1.07–1.92)	0.016			1.19 (0.88–1.61)	0.258
Subgrouping 2^§^	1.89 (1.59–2.26)	<0.001	1.85 (1.53–2.24)	<0.001		

Abbreviation: HR, hazard ratio; CI, confidence interval; LC, liver cirrhosis; TACE, transarterial chemoembolization

* Female *vs*. male

^†^ TACE *vs*. surgical resection; Analysis was performed only for the patients who underwent TACE or surgical resection as an initial HCC treatment.

^‡^ B1 *vs*. A1 + A2 + A3

^§^ A3 + B1 *vs*. A1 + A2

^1^ Multivariate analysis regarding subgrouping 1

^2^ Multivariate analysis regarding subgrouping 2.

To confirm the superior OS with surgical resection over TACE in each subgroup, we additionally performed Cox regression analysis for each subgroup. For all subgroups, surgical resection performed superior OS over TACE as follows: A1 (HR 2.5; 95% CI 1.86–3.36; *P*<0.0001), A2 (HR 3.42; 95% CI 1.48–7.94; *P* = 0.004), A3 (HR 3.0; 95% CI 2.08–4.33; *P*<0.0001), and B1 (HR 2.42; 95% CI 1.15–5.07; *P* = 0.019).

Even, after IPW, subgrouping 2 (HR 1.88; 95% CI 1.52–2.34; *P*<0.001) was still a significant predictive factor for OS, which was not in subgrouping 1 (HR 1.19; 95% CI 0.88–1.61; *P* = 0.261) (Table 3).

**Table 3:** Assessment of discrimination ability of respective subgroupings.

Subgrouping	AIC	C-index	HR (95% CI)
Subgrouping 1 (A1 + A2 + A3 *vs*. B1)	5801.8	0.618 (0.593–0.643)	1.19 (0.88–1.61)
Subgrouping 2 (A1 + A2 *vs*. A3 + B1)	5765.0	0.651 (0.625–0.677)	1.85 (1.53–2.24)
**After IPW**			
Subgrouping 1 (A1 + A2 + A3 *vs*. B1)	5766.3	0.619 (0.594–0.643)	1.19 (0.88–1.61)
Subgrouping 2 (A1 + A2 *vs*. A3 + B1)	5727.2	0.652 (0.626–0.678)	1.88 (1.52–2.34)

Abbreviation: AIC, Akaike information criterion; HR, hazard ratio; CI, confidence interval; IPW, inverse probability weighting.

### Assessment of discrimination ability of respective subgroupings

As compared to subgrouping 1, subgrouping 2 showed superior discrimination function with the lower AIC value (5801.8 *vs*. 5765.0) and the higher c-statistic (0.618 *vs*.0.651) (Table 3). Moreover, even after IPW, subgrouping 2 still had the lower AIC value (5766.3 *vs*. 5727.2) and the higher c-statistic (0.619 *vs*.0.652).

**Fig 6:**
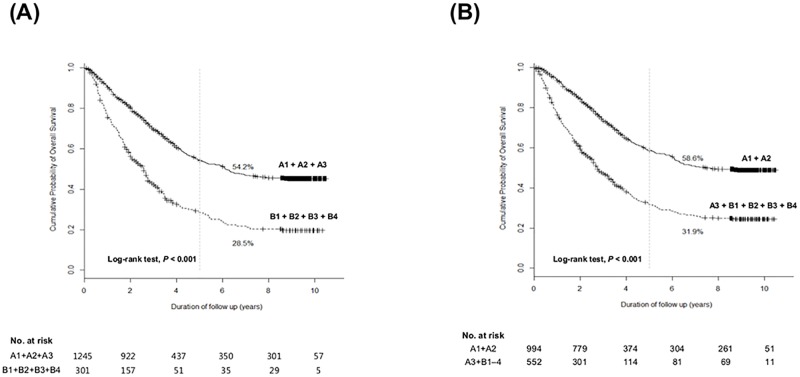
Cumulative overall survival rate of each subgrouping when other intermediate tumors (B2–B4) were taken into consideration. (A) Overall survival rates according to subgrouping ‘A1–A3 *vs*. B1–B4’ (B) Overall survival rates according to subgrouping ‘A1–A2 *vs*. A3–B4’.

We performed additional survival analysis taking HCC patients with other intermediate stages (B2, B3 and B4; n = 205) into consideration. As compared to subgrouping ‘A1–A3 *vs*. B1–B4’ (Fig 6A; HR 2.26; 95% CI 1.92–2.67; *P*<0.0001), subgrouping ‘A1–A2 *vs*. A3–B4’ (Fig 6B; HR 2.31; 95% CI 1.99–2.68; *P*<0.0001) also showed superior discrimination function on OS with the lower AIC value (9599.9 *vs*. 9563.7) and the higher c-statistic (0.574 *vs*.0.613).

## Discussion

In this study, we have analyzed the identity of single large (>5 cm) HCC, as an aspect of BCLC subclassification. Single tumor is classified into early stage (stage A) in the BCLC staging system without any consideration of tumor size [10]. However, in this study, single large tumor showed significantly worse survival, which indicate that single tumor should be differently staged according to their tumor size. When single large tumor was grouped as intermediate stage tumor, it showed superior survival prediction than when it was grouped as early stage tumor. This indicates that in terms of prognosis a single large tumor should be considered as intermediate stage.

Our findings are consistent with several studies which showed high risk of tumor recurrence and poor OS for a tumor exceeding 5 cm [20–24]. Although some studies have reported similar survival between single large HCCs and single small HCCs [25, 26], they were composed of well-selected patients who have received resection. However, most recent studies revealed that solitary large HCC should be classified at least as intermediate stage HCC [27, 28].

Tumor size is associated with the presence of microvascular invasion and with the histologic grade of HCC [29, 30]. One study reported that the incidence of microscopic vascular invasion was almost twice as high in tumors larger than 5 cm (61%) as in smaller tumors (32%). The incidence continued to rise even in tumors larger than 10 cm [30]. Patient with microvascular invasion shows significantly higher recurrence and shows a worse OS [31, 32]. In case of liver transplantation, tumor size is an important factor in single tumor. Tumors only smaller than 5 cm are considered eligible for transplantation in the Milan criteria, as large size tumor are associated with higher risk of recurrence [33]. As poor outcome of single large tumor can be explained by microscopic vascular invasion or poorer tumor grade, subgrouping of single large tumor according to microscopic vascular invasion or poorer tumor grade can be considered. However, microscopic vascular invasion or tumor grade cannot be easily identified by current dynamic computed tomography or magnetic resonance imaging. Presently, as an alternative but acceptable surrogate, one has to rely on tumor size. One major aim of tumor staging is to identify subgroup which shows similar survival. In this aspect, single large tumor and single small tumor should be differently staged to each other.

We have also performed IPW to balance the covariates including sex, age, the etiology of HCC, and the presence of LC. Even after IPW, subgrouping 2 showed superior survival prediction over subgrouping 1. Moreover, in subgroup analysis, subgrouping 2 showed superior survival prediction over subgrouping 1 regardless of initial treatment modality. Especially, for the patients who underwent surgical resection as an initial treatment, subgrouping 1 could not discriminate OS between BCLC A and BCLC B1 stage. However, subgrouping 2 significantly discriminated OS during the follow-up period.

HCC patients who underwent TACE experienced poor survival rate during the follow-up period compared to those who underwent surgical resection as an initial treatment, which means single large HCC should not be regarded as a contraindication for surgical resection. Although single large tumor should be considered as intermediate stage in terms of prognosis, resection might be the first line treatment option for the patients without portal hypertension. If the patient has portal hypertension who is ineligible for surgical resection, TACE might be an alternative treatment option with comparable OS outcome to that of surgical resection [34].

Our study has several limitations that need to be considered. This is a retrospective cohort study with inherent limitations. The decision of treatment was selected by a respective physician in each center, thus, unidentifiable bias may be present in the selection of treatment for each patient. Therefore, although our data suggest that resection can be preferred over TACE for a single large tumor, a prospective study is needed to definitely say resection can be preferred the option. This study was conducted in Korea, where most of tumor are hepatitis B virus (HBV)-related HCCs. HCC shows different characteristics according to the underlying disease [35], and potent nucleos(t)ide analogues are available to preserve liver function during HCC treatment. Therefore, generalizability should be validated in area where major etiology of HCC is not HBV. The presence of LC was not a significant factor predicting OS in multivariate Cox regression analysis. Most of the patients who had LC were with Child-Pugh class A, which might lead to little effect on OS.

Our study has strengths in that it is a large scale study with long-term follow-up data, which enrolled consecutive patients to minimize selection bias. IPW was also used to minimize bias. The aim of cancer staging is to estimate a person’s prognosis, to help plan the appropriate treatment, and to provide common terminology for exchanging information [36]. To further fulfill the required purpose of cancer staging system, our data calls for the refinement of BCLC staging system to better predict prognosis, to better help select the appropriate treatment, and to better give common terminology for exchange information.

This study showed that subgrouping of single large tumor to intermediate stage better stratified patient prognosis, indicating that single large tumor should be considered as an intermediate stage in terms of prognosis. In the aspect of treatment, resection might be the first line treatment option for a single large tumor, although prospective validations are needed.

## Abbreviations

HBV: hepatitis B virus
HCC: hepatocellular carcinoma
BCLC: Barcelona Liver Clinic
OS: overall survival
AUC: area under the curve
AIC: Akaike information criterion
TACE: transarterial chemoembolization
PEI: percutaneous ethanol injection
RFA: radiofrequency ablation
HR: hazard ratio
CI: confidence interval
AFP: alpha-fetoprotein
AASLD: American Association for the Study of Liver Diseases
RECIST: Response Evaluation Criteria in Solid Tumors
CP: Child-Pugh
ALT: alanine transaminase
AJCC: American Joint Committee on Cancer
AST: aspartate transaminase
APASL: Asia-Pacific Association for the Study of the Liver
EASL: European Association for the Study of the Liver
HBeAg: hepatitis B e antigen
MELD: Model for End-Stage Liver Disease
